# Development of a biomechanical model for dynamic occlusal stress analysis

**DOI:** 10.1038/s41368-021-00133-5

**Published:** 2021-09-08

**Authors:** Zheng Duanmu, Lu Liu, Qi Deng, Yuanyuan Ren, Meiqing Wang

**Affiliations:** 1 Key Laboratory of the Ministry of Education for Optoelectronic Measurement Technology and Instrument, Beijing Information Science and Technology University, Beijing, China; 2 Department of Oral Anatomy and Physiology and TMD, School of Stomatology, Air Force Medical University, Xi’an, China

**Keywords:** Biomedical engineering, Bone quality and biomechanics

## Abstract

The use of traditional finite element method (FEM) in occlusal stress analysis is limited due to the complexity of musculature simulation. The present purpose was to develop a displacement boundary condition (DBC)-FEM, which evaded the muscle factor, to predict the dynamic occlusal stress. The geometry of the DBC-FEM was developed based on the scanned plastic casts obtained from a volunteer. The electrognathographic and video recorded jaw positional messages were adopted to analyze the dynamic occlusal stress. The volunteer exhibited asymmetrical lateral movements, so that the occlusal stress was further analyzed by using the parameters obtained from the right-side eccentric movement, which was 6.9 mm long, in the stress task of the left-side eccentric movement, which was 4.1 mm long. Further, virtual occlusion modification was performed by using the carving tool software aiming to improve the occlusal morphology at the loading sites. T-Scan Occlusal System was used as a control of the in vivo detection for the location and strength of the occlusal contacts. Data obtained from the calculation using the present developed DBC-FEM indicated that the stress distribution on the dental surface changed dynamically with the occlusal contacts. Consistent with the T-Scan recordings, the right-side molars always showed contacts and higher levels of stress. Replacing the left-side eccentric movement trace by the right-side one enhanced the simulated stress on the right-side molars while modification of the right-side molars reduced the simulated stress. The present DBC-FEM offers a creative approach for pragmatic occlusion stress prediction.

## Introduction

Occlusion is defined as: (i) the act or process of closure or of being closed or shut off; (ii) the static relationship between the incising, masticating surfaces of the maxillary or mandibular teeth or tooth analogues.^1^ The primary function of occlusion is to chew up foods with exact tooth contacts and large forces of food-crushing.^2^ Overloading may lead to tooth wear,^3–5^ fatigue,^6–9^ cervical lesions,^10–12^ and cracks from the contact zone at the occlusal surface of the nature teeth.^13,14^ Hence, dental stress under occlusal loading has been largely reported, especially in the field of restorations’ design.^15–17^ Researches indicated that food stiffness has a slight impact on the stress distribution of the restored and sound teeth because the stress distribution does not differ particularly in the same geometrical configuration of cavity with varying stiffness of food.^18^ The impact of dental geometry is then of significance because it determines the occlusion contact areas where the mastication force is focused.^19,20^ The contact between tooth surface and food bolus alter dynamically during chewing, meaning that the masticatory forces on dentition constantly change in direction, intensity, and point of application.^21^ The uneven occlusal contact surface divides the loading following the principles of force decomposition that can be transferred to roots and then the periodontal tissues.^22^ The loading messages are picked up by periodontal mechanoreceptors. By activating the periodontal-trigeminal mesencephalic nucleus-trigeminal motor nucleus circuit, occlusion modifies the jaw muscles’ activity.^23^ About 85% of the muscular activity necessary to chew is peripherally induced, that means, the contact stress message of the uneven occlusion takes a pivotal role in feedback controlling of the jaw muscles activity during chewing function.^24^ Obviously, dental stress during simulated centric and eccentric clenching is meaningful in view of function and dysfunction of masticatory system, yet the stressing regularity during centric and eccentric clenching remains undetermined.

There are the largest contacts in the maximum intercuspal occlusion situation, while there are fewer contacts in the protrusion and lateral excursive occlusion.^25^ Dynamic occlusal contacts had been discussed greatly in literatures for its interferential role.^26–28^ The posterior contacts during protrusion and the balancing side contacts during lateral excursion had been proposed as the interferences because they might be the causes of temporomandibular disorders (TMD).^28^ However, diverse reports are documented regarding the view point that the balancing interferences are harmful to oral function. Some researchers indicated no differences in the frequency of the interferences between the TMD patients and healthy peoples.^29–31^ The limited strength information of the interferential contacts might be one of the explanations for that inconsistency. In literature, efforts had made to describe the contact features of the occlusion with strength message. For example, T-Scan system provides locational loading information with time-dependent strength relative values. An inserted transducer recorded the detection procedure of dentitions. Even though thin to 60 μm, the transducer affects the test accuracy.^32^ Further, the uniformity of the transducer thickness prevents the T-scan from a real contact provider.

Direct measurements of tooth contacts and forces are difficult. Biomechanical models are better for understanding the relationship between occlusion and function.^2^ In literature, the virtual simulation tool of finite element method has been widely used in mandibular lever analysis, most often on tooth or TMJ condyles.^19,33–36^ In the reported finite element models (FEM), the stress analysis based on the muscle force simulation is often simplified as specific concentrate forces at particular bite locations. The jaw movements are relatively small and directional perplexing that are under fine neural control.^37^ For example, muscles with a generally vertical orientation are responsible for fine horizontal regulation of movement and stabilization.^38^ That means each muscle can influence more than one degree of freedom. The schematic representation of a single line of action is incomplete, so more realistic loadings are then required for bite simulation.^39^

It is indicated that stress analysis can be performed based on muscle liked concentrated force and the defined displacement boundary condition.^40^ Using the recorded mandibular displacement as the boundary condition, a reformative FEM, termed the displacement boundary condition finite element model (DBC-FEM)^41^ could be developed where the muscular forces are no longer necessary to analyze occlusal stress. The DBC-FEM model is an explicit scheme that efficiently solves highly nonlinear problems, especially when dealing with complex contacts and large deformations.^42^ Giving a uniform linear motion, the larger a contact size is, the smaller the contact stress will be. When a tooth exposes its different sites to have occlusal contacts with the opposing tooth, various stresses came out according to the contact size of the sites. Then, the stress at the contact sites could be obtained based on the contact size following the penalty algorithms^43,44^ as DBC-FEM provides.

This paper developed a DBC-FEM by using the jaw kinematic three-dimensional movement recordings. The location and size of the dynamic occlusion contact were computed, and the derived occlusal stress was analyzed. The purpose was to explore a creative approach for occlusion stress prediction without either interrupting nature behaviors of dental occlusion or simulation of muscles force.

## Results

The data from EGN recordings are presented in Fig. 4c and Supplemental Tables 1–4. The volunteer showed asymmetrical lateral excursive movements, more prominent on the right side than the left side, which were 6.9 and 4.1 mm, respectively.

### Occlusion stress in the simulated centric and eccentric movements—obtaining from the tasks in section 1

**Fig. 1:**
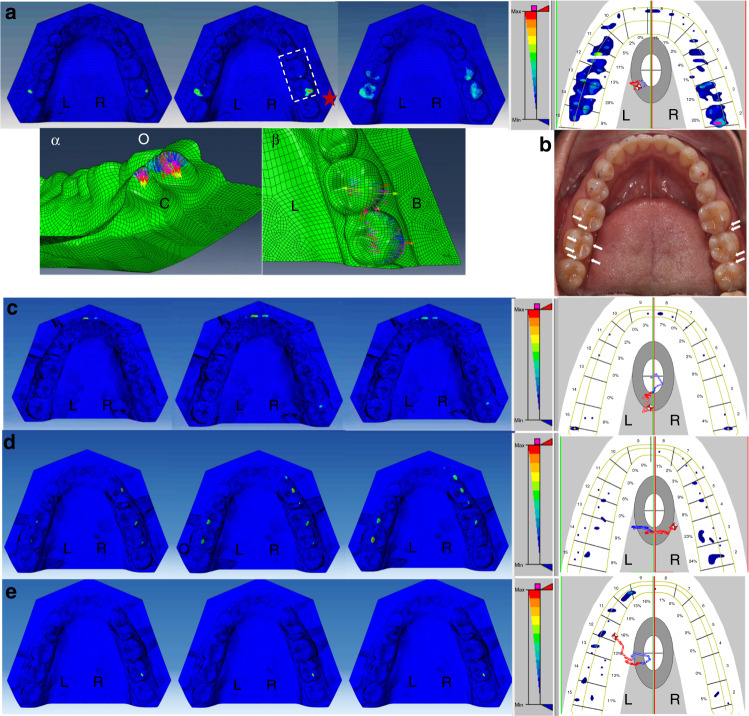
The occlusion stress representation at initial (left), middle (middle), and end (right) stage of the simulated contact and the representative T-scan occlusion recordings of the four defined dynamic tasks. The typical locations of the maximum stress are marked with red stars in each panel. **a** Centric closing; the image of the local sites with the highest values of the maximum stress were squared and magnified in panels α the bottom view, and β the top view. **b** the articulating papers measurements in centric occlusion. The occlusal imprints are scatter in distribution and slight in strength although the voluntary maximal effect was required. The blue and red imprints merged well as the arrows indicate. **c** The centric to protrusion; **d** Right-side lateral extension; **e** Left-side lateral extension. O, occlusion side; C, bottom side; L, lingual side; B, buccal side

The DBC-FEM model successfully revealed the shear and vertical stress at different stages of each task. Generally, the stress value changed when the contact size increased or decreased in the dynamic tasks. Three images representing the occlusal maximum stress value at the lowest, middle, and largest level in each task are presented in Fig. 1.

#### The centric closing task (Fig. 1a)

The highest maximum stress value was 117 GPa, located at the right-side second molar. All the maximum stress values were increased when the mandibular dentition was simulated closing to the maxillary dentition from the early to the later closing stage. Similarity was noticed when the red and blue articulating papers (Tianjin Shengshili Dental Materials Factory, Tianjin, China) were used separately during the maximal voluntary biting in centric occlusion. The two records merged well (Fig. 1b), although no speckle occlusal imprints were obtained after several times attempts. The T-Scan graph presented the contacts that were located at the bilateral molar regions, coincidental with the simulation in the DBC-FEM model.

#### The centric to protrusion task (Fig. 1c)

The maximum stress was initially located at the bilateral molar regions but gradually at the region of the central incisors during the protruding task. In the end stage of protrusion, the stress concentration was noticed at the right-side third molar. The highest value of the maximum stress was 110 GPa, which appeared at the end stage of protrusion, located at the incisors. The T-Scan graph showed similarities and the contacts displayed at bilateral incisor and molar regions. The contact at higher level strength appeared at the right-side third molar in T-Scan, identical with simulation in the DBC-FEM model.

#### The centric to lateral extension task (Fig. 1d, e)

When simulating the mandible to excurse to the right side, the maximum stress was observed on not only the right-side canine, the premolars, and the first molar, but also on the left-side first and second molars at the late stage of the task. The highest value of the maximum stress was 53.3 GPa, which appeared at the end stage of the right-side lateral excursion, located at the left-side second molar. The T-Scan graph showed quite a similarity. The contacts appeared at the canine, premolar, and molar region of the right side and the molar region of the left side. When the mandible was simulated to excurse to the left side, the maximum stress was observed only at the right-side second molar. No maximum stress was observable at the left-side arch. The highest value of the maximum stress was 5.48 GPa, which appeared at the end stage of the left-side excursion, located at the right-side second molar. However, the T-Scan graph showed contacts at the left-side arch and at the right-side molar.

### Occlusion stress in the simulated symmetrical lateral excursive movements—obtaining from the tasks in section 2

**Fig. 2:**
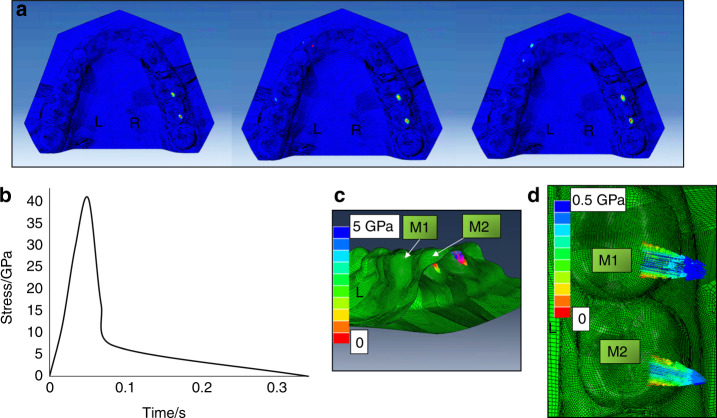
The occlusion stress representation for the simulated left-side lateral excursion movement according to the right-side lateral excursion recording. **a** The stress distribution at the initial (left), middle (middle), and end (right) stage of the simulated left-side lateral excursion movement; **b** The time–stress curve of the maximum stress at the right-side second molar. **c** The vertical stress. **d** The shear stress. M1 the right-side first molar, M2 the right-side second molar

Recordings from EGN demonstrated that the volunteer had a shorter left-side excursive range, which was 4.1 mm leftward and 1.9 mm downward, than the right-side excursive range, which was 6.9 mm rightward and 6.6 mm downward. When simulating the left-side excursion task according to the right-side lateral excursion recordings, the maximum stress on the right-side first and second molars turned more significant (Fig. 2a). There was maximum stress on the left-side first molar at the middle stage. The maximum stress happened on the left-side canine and the first premolar at the end stage. The values of the maximum stress from the initial stage to the end stage are shown in Fig. 2b. The highest value of the maximum stress was on the right-side second molar, which was 40.7 GPa. The normal and shear stress of that site were 5 and 0.5 GPa, respectively (Fig. 2c, d). When simulating the right-side excursion task according to the left-side lateral excursion recordings, no maximum stress showed on the whole arch.

### Occlusion stress after the virtual tooth morphological modification—obtaining from the tasks in section 3

**Fig. 3:**
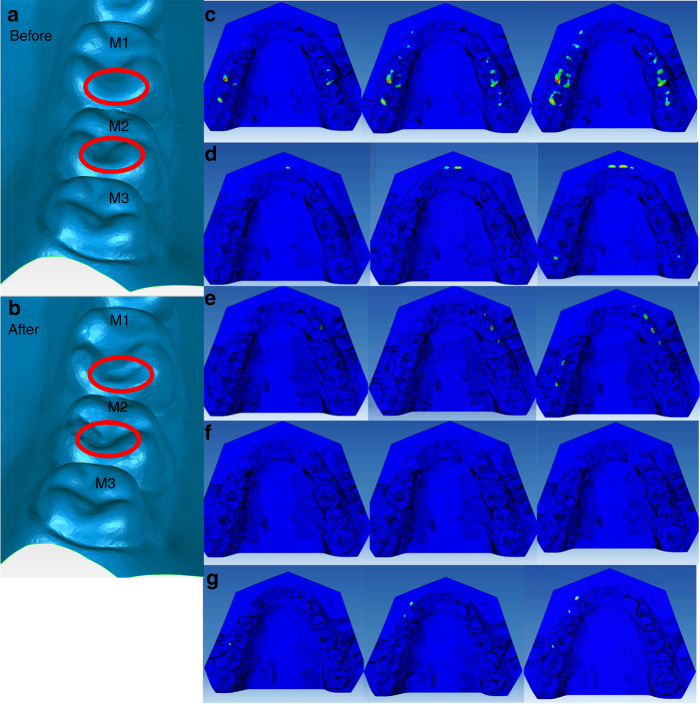
The geometry of the right-side molars (M1-3) before (**a**) and after (**b**) the virtual tooth morphological modification, and the occlusion stress distributions after in the virtual tooth modification in the tasks of Section 1, i.e., centric closing (**c**), the protrusion (**d**), the right-side excursion (**e**) and left-side excursion (**f**), and in the task of Section 2 (**g**)

Virtual teeth morphological modification was performed on the lingual ridge of the distal buccal cusps of the right-side mandibular first molar and second molar (Fig. 3c). There were contacts during the left-side lateral excursion (Fig. 1) and the simulated symmetrical left-side lateral excursion (Fig. 2). After the virtual morphological modification, the maximum stress in the simulated centric task was distributed with more symmetry, wider, and broader on the arch (Fig. 3c). There was maximum stress located at the premolar region, which was not the case before modification (Figs. 3d, 1a). The highest value of the maximum stress was 66.8 GPa, located at the left second molar after virtual modification.

In the centric to protrusion task, there was still maximum stress at the central incisors region and the right third molars. Unexpectedly, there appeared contact on the left-side third molar at the end stage of the task (Fig. 3e). In the centric to right-side lateral extension task, there was still maximum stress on the right-side canine and the first and second premolars, and also on the left-side first and second molars, but no more on the right-side first molar (Fig. 3e). However, in the centric to left-side lateral extension task, no maximum stress was observed (Fig. 3f). When simulating the left-side excursion task according to the right-side lateral excursion recordings, the maximum stress occurred on the left-side canine and the first premolar region (Fig. 3g).

## Discussion

Biomechanics analysis helps understand the structure and function of biological systems and the forces on and displacements of the dental occlusion.^2^ In this work, we developed a method to create a DBC-FEM that makes it possible to analyze the dynamic occlusal stress at the real sense contact sites according to the EGN and video recordings. Taking the recorded kinematical data as the boundary condition makes it unnecessary to simulate the complex muscular forces. The penalty formulation with finite sliding could prevent the unlimited permeated contacts, which is impossible in real conditions. With this DBC-FEM, we analyzed occlusion stress on the fluctuation surface of the mandibular dentition during nature closing, protrusive, and eccentric movements. In line with the lateral literature,^36,45^ the highest maximum value of stress was in terms of GPa unit. Our present DBC-FEM model provides a new approach to evaluate occlusal stress and virtual occlusal correction in daily clinical practices or research, which helps correct clinical misperceptions and hopefully inform better patient care.

Functional studies considering the kinematics of teeth are essential to understand biomechanics and interpret morphological adaptation of teeth.^35^ The present volunteer did not know that she has a problem excursing her mandible to the left side as precisely as what she did for the right side until she attended the present test. In the test process of the left-side excursion task, she tried her best to make the left-side teeth in contact with experimental requirements. By using her DBC-FEM for analyzing the occlusal stress, we revealed that the highest value of the maximum stress was located on her right-side molars when performing the left-side lateral excursion. It should be those right-side contacts, or the balance side interference, that prevented the teeth of the left side or the working side from contact. We performed the left-side lateral excursive movement simulation with the mirror trace of the right side. The result showed that the right-side interference contacts got heavier (Fig. 2). Such heavy contacts should be harmful to the masticatory organs. The potential harmfulness of the serious contacts should be why she moved to the left side with a larger vertical dimension but shorter lateral extension than moving to the right side. Such a successful protective compensation in the mandibular movement explains, at least partially, her free of disordered symptoms. Avoidance of heavy occlusion contact via alteration of mandibular movement pattern is, thus, an important design of the masticatory system. With such mechanisms, the masticatory system is adapted to complex changes in the dental occlusion and exhibits a high level of damage tolerance.^2^

The biomechanical effects of occlusal loads on teeth during clenching and mastication and the transfer of occlusal forces have been primarily reported in the literature.^46,47^ In a chewing simulation study, occlusal contact stiffness was the key point substantially affecting maximum contact force.^48^ Clenching of molars and masticating morsels of high elastic moduli evoke considerable stress concentrations in the occlusal enamel of these teeth. While masticating a morsel of low elastic modulus, which conforms to the occlusal surfaces of teeth, creates considerable stresses in the cervical portion of the lingual wall of the mandibular molar.^47^ Even though, the dental stress distribution patterns are more likely to be affected by loading direction and position.^35^ Loads oriented normal to the tooth axis, such as that in subjects with balanced occlusion, are better distributed to the supporting tissues, thus was believed capable of avoiding tooth bending and stress concentrations.^12^ Instable occlusion, on the other hand, is linked with tooth-damage-inducible occlusal forces. With many masticatory cycles, the unstable occlusion could be damage-inducible and cause tooth fracture.^49^ A compelling feature of the splitting load relation is its explicit dependence on key geometrical dimensions.^50^ Some anatomic occlusion forms have a higher fracture potential, such as the nonfunctional cusps versus functional cusps due to the wedging effect of the cusp–fossa.^13^ Grooves and fissures on the occlusal surface had been taken as critical locations of cracks because tensile stresses on a FEM model were concentrated at these features.^35^ It is then essential in clinical practice to identify the contact areas to estimate how the chewing force is distributed in the tooth and in the supporting structure^51^ to provide an optimal occlusion load to ensure the long-term success of dental treatment.

In addition to dental healthy, occlusion has an impact on temporomandibular joints (TMJs), jaw muscles^2^ and even cervical and trunk muscles.^52^ Experimental occlusal interference in animals could cause jaw muscle damage, fatigue, and pain.^53–55^ During the mastication process, the occlusion and the TMJs suffer the loading from jaw muscles’ contraction, which is originally active to chew up foods. The periodontal proprioceptors will pick up the message of occlusion loadings and then modify the jaw muscles activity via periodontal-muscle reflex mechanisms. The dynamic occlusal stress is then worth recording and measuring. However, as far as our extension, in literature, there are still no dynamic occlusal stress evaluation devices or systems in view of oral function and dysfunction.^56^ Clinical measurements like laser scanner, occlusal stress detector, electromyograph, and mandibular movement recorder are generally used in an independent pattern for diagnosing occlusal functions.^57^ None of them directly provide the stress information. The clinical loading assessment relies primarily on the doctor’s personal experience and strong empirical operation by using a single bite size evaluation to the stress tester, which lacks quantitative indicators.^58^ Our DBC-FEM method brings about a new insight for dynamic occlusion stress assessment. With our developed method, the occlusion stress can be analyzed, and the virtual occlusion modification could be performed to achieve satisfactory occlusion function.

As far as our extension, this work is the first one in the literature that describes the movements setting in three directions and combined using dimensions and rotations based on the EGN and video recordings. The EGN and videos were not synchronously recorded due to the limitations of the recording technique. We segmented the EGN and videos recordings by time so that the video angle measurements could match the ENG displacement measurements. Even though the system errors existed, which contributed, at least partially, to the minor differences of the present DBC-FEM data from T-Scan recordings. However, the minor differences may also come from the T-Scan transducer’s obstacle interferences, which were more predominant during protrusion and lateral excursions. The even thickness of the transducer prevented the freedom of the dynamic tasks and increased the possibility of systemic errors. The system errors have to be taken into account, especially when aiming for occlusal correction. More complex composite three-dimensional solid structure and nonlinear material models are expected to develop. The high simulation model studies are expected to be conducted, for example, the kinematics of the simulated chewing tasks and linear shell element structure for occlusal simulation.

## Conclusion

In summary, taking the mandibular kinematical parameters as the boundary condition, the present contact derived from DBC-FEM brings about a creative approach for occlusion stress prediction. Importantly, our DBC-FEM makes it practical to correct occlusion biomechanics through virtual morphological modification. It will be helpful in occlusion-related operations such as computer aided design and computer aided manufacturing (CAD/CAM) for denture processing.

## Materials and methods

This study collected information from a 32-years-old Chinese female volunteer. She had no symptoms of oral dysfunction, such as that observed in patients with temporomandibular disorders (TMDs). She declared no known bruxism. She had no tooth surface lesions such as severe tooth wear, cervical lesions, and caries, and her periodontal condition was healthy as examined by one of the authors (MQ). She understood the task well because she is a dental nurse. She had 30 permanent teeth arranged in morphological normal. The measurements were performed by one of the authors (DQ). All the procedures were conducted according to established ethical guidelines with written consent. This study was approved by the Human Experiment Committee, College of Stomatology, the Fourth Military Medical University (Ethics Certificate No: IRB-REV-2015031).

### Dentition reconstruction

**Fig. 4:**
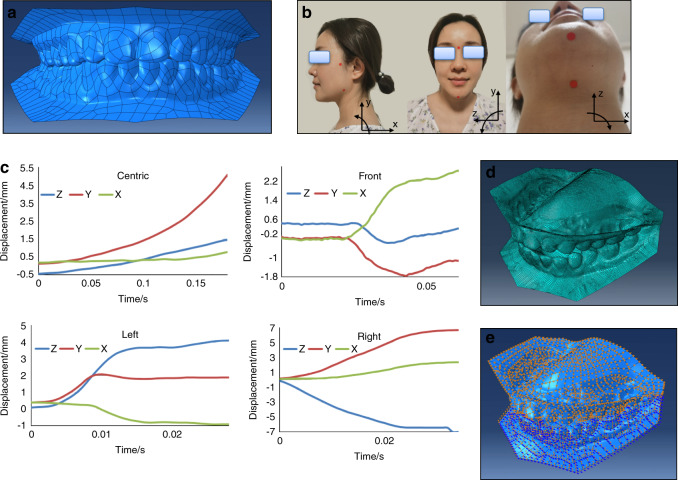
Development and application of the displacement boundary condition finite element model (DBC-FEM) and the jaw rotation movements recording methods. **a** The full reconstructed 3D dentition surface models positioned at centric occlusion. **b** Diagram for jaw rotation video recording. The pogonion, gonion, nasion, hyoid bone, and root of zygomatic process (RZP), as indicated by red spots, were taken as the osteological landmarks. Video of the jaw rotation movements was recorded in sagittal (*x-* and *y*-axis), frontal (*y*- and *z*-axis), and horizontal (*x*- and *z*-axis) planes, separately. **c** The mandibular movements of three plane decomposition originated from ENG recordings. **d** The dentitions mesh elements. **e** The displacement boundary conditions. The blue arrows represent rotational loadings, and the orange ones represent displacement loadings

Plastic casts were obtained and then scanned using a dental scanner (3Shape R750, 3Shape, Denmark). The 3D digital model was created using 3Shape software (3Shape ScanItManagerTM, 1.7.1.0, 3Shape, Denmark) and smoothed by using Model Preparation software (3Shape OrthoAnalyzerTM, 1.7.1.0, 3Shape, Denmark). The static and dynamic noises were removed through mean filtering from raw data. The noise was further removed utilizing the gray histogram analysis and the threshold value. The entire dentition models were reconstructed with upper 589 and lower 509 pieces curved surfaces after smoothing (Fig. 4a).

### Electrognathograph (EGN)

The volunteer was trained before testing. The mandibular movements were recorded by EGN and BioPAK system software (both by Bioresearch Associates, Inc., Milwaukee, WI, USA).^41^ Briefly, a magnet was attached to the labial surface of the mandibular central incisors without interfering with any movements of the mandible. The sensor array was fixed on the subject’s head, as we previously reported.^36^ The measurements recorded the nature close from the rest position to the centric occlusion position in three dimensions. Followed by protrusion and the right- and left-side lateral excursion movement. All of the movements were started from the intercuspal position (ICP). During the processes of the movements, the maxillary and mandibular teeth were required to be kept in touch. The sampling frequency was 1 000 Hz. The displacing amplitudes were automatically calculated and displayed by the software (BioPAK software Version 8.1, BioResearch Associates, Inc., Milwaukee, WI, USA). During the whole testing process, the subject sat upright in a chair, keeping the eyes on a point at eye level two meters away as required.

### Video of the jaw rotation movements

The rotation of the jaw during centric and eccentric movements were recorded by a video camera (Canon EOS 6D, Canon Inc., USA). The locations of the osteological landmarks were pogonion, gonion, nasion, hyoid bone, and root of the zygomatic process (RZP) (Fig. 4b). When the mandibular rotational messages were extracted from the video recordings for the 3D motion coordinates deposition, different pairs of markers were used. One of the markers in each pair was fixed and the other was movable. RZP was taken as the fixed and gonion as the movable for saggital view, nasion as fixed and pogonion as movable for frontal view, and hyoid bone as fixed and pogonion as movable for horizontal view. By taking the video of the centric and eccentric movements, the 3D motion coordinates can be deposited into sagittal (with RZP and gonion), frontal (with nasion and pogonion), and horizontal (with hyoid bone and pogonion) planes, each with two directions displacements and one rotation. The displacement curves of different occlusions are shown in Fig. 4c. The rotated angles of each plane are shown in Table 1.

**Table 1:** The rotated angles of each plane

Recording plane	Centric task	Eccentric tasks		
	Nature closing	Protrusion	Right-side lateral excursion	Left-side lateral excursion
Sagittal	0.104	0.111	0.084	0.052
Frontal	0.001	0.002	0.001	0.002
Horizontal	0.003	0.001	0.471	0.179

### DBC-FEM modeling

The material property of this research was assumed to be homogeneous, isotropic, and linear elastic. The whole dentition is processing as enamel shell elements. The elastic modulus for enamel was defined as 84 GPa with a Poisson’s ratio of 0.3.^36^ The total 3D occlusion model was meshed in Abaqus software (Version 14.0, Dassault Systemes, Co., Velizy-Villacoublay, FRANCE) with 60 345 nodes and 60,309 elements, which include 943 linear triangles and 59 336 linear quadrilaterals (Fig. 4d). The displacement and angle recordings were used as merged data by taking the initial centric occlusion position as the common reference frames. The accuracy of the two recordings were different, more precise in ENG recordings than video recordings. We segmented the recordings by time scales, and the data from the same segment was merged. The merged datasets, containing 18, 61, 29, and 35 segments separately for the four tasks of the centric closing, protrusion and left-side and right-side lateral excursion, were delivered to the DBC-FEM model for dynamic stress analysis.

### The boundary condition for the stress analysis using DBC-FEM

The mandibular dentition was simulated to move under the fixed maxillary dentition. The movement was set as three directional displacements and rotations based on the EGN and video recordings (Fig. 4e). The displacements can be captured by tracing a magnetic point in the ENG, and the rotational angles can be captured by tracing the markers in the video recordings. The volunteer first kept in the initial occlusal gesture with mouth closed, and then was required to start the four moving tasks. The displacements data from EGN and video derived from the same task were matched by time scales. The displacement and angle data were merging analyzed by taking the initial gesture as the common reference frames. The accuracy of ENG was higher than video, but with the time scales as labels, the system errors were diminished to minimal. The contacts between the pairs of the maxillary and mandibular dentitions were set with friction coefficient 0.1 and no elastic slip on the interactions.^59^ The contact stress was calculated proportionally according to the occlusal contact areas at each material point of the model following penalty algorithms.

### The simulated tasks

In total three sets of tasks were simulated.

Section 1: Four simulated tasks were centric movement, which was the nature closing to the centric occlusion position, and eccentric movement, which included protrusion from the centric position to the edge-to-edge relation, and lateral excursion from the centric position to the right and left side, separately, with the mandibular buccal cusps directly under the maxillary buccal cusps of the molars.

Section 2: Applying the mirror trace of the right-side lateral excursion movement to the left-side lateral excursion movement to achieve complete symmetrical lateral tasks. The left-side lateral excursion movement was also applied to the right side for reference.

Section 3: Applying a virtual removal of the lateral balance interferential contacts on the right-side first and second molars. Then the rehabilitated model was applied with the four simulated tasks as that in section 1 and the mirror simulated task as in section 2.

### Stress analysis

The dynamic contact sizes were calculated to obtain the contact derived from maximum principal stress on the mandibular dentition. The stress was analyzed based on the decomposed normal and shear components in the environment of the Abaqus software (Version 14.0, Dassault Systemes, Co., Velizy-Villacoublay, FRANCE). The locations and the values of the maximum stress in each task were presented.

### T-can

The location and strength of the occlusion contacts recorded with the T-Scan III occlusion analysis system (Tekscan, Inc., Boston, MA, USA)^34^ were taken as contrasts. The thickness of the sensor was 60 μm. The time-dependent number and relative strength level of occlusal contacts were displayed as color contour images. The system set the force threshold before testing according to the instrumental introduction.

## Supplementary information

supplemental Table+1-4

## References

[1] Aidsman IK (1977). Glossary of prosthodontic terms. J. Prosthet. Dent..

[2] Peck C (2016). Biomechanics of occlusion—implications for oral rehabilitation. J. Oral. Rehabil..

[3] Ghazal M, Yang B, Ludwig K, Kern M (2008). Two-body wear of resin and ceramic denture teeth in comparison to human enamel. Dent. Mater..

[4] Lambrechts, P., Debels, E. Van Landuyt, K., Peumans, M. & Van Meerbeek, B. How to simulate wear?: overview of existing methods. *Dent. Mater.* **22**, 693–701 (2006). 10.1016/j.dental.2006.02.004 16712913

[5] Stober T, Lutz T, Gilde H, Rammelsberg P (2006). Wear of resin denture teeth by two-body contact. Dent. Mater..

[6] Blatz, M. B., Oppes, S., Chiche, G., Holst, S. & Sadan, A. Influence of cementation technique on fracture strength and leakage of alumina all-ceramic crowns after cyclic loading. Quintessence international 39.1 (2008). 18551213

[7] Naumann M (2009). Influence of test parameters on in vitro fracture resistance of post‐endodontic restorations: a structured review. J. Oral. Rehabil..

[8] Ohlmann, B. et al. Fracture-load values of all-ceramic cantilevered FPDs with different framework designs. *Int. J. Prosthodont.* **22**.1 (2009). 19260427

[9] Zhang Y, Sailer I, Lawn BR (2013). Fatigue of dental ceramics. J. Dent..

[10] Dejak B, Mlotkowski A, Romanowicz M (2005). Finite element analysis of mechanism of cervical lesion formation in simulated molars during mastication and parafunction. J. Prosthet. Dent..

[11] Takehara J, Takano T, Akhter R, Morita M (2008). Correlations of noncarious cervical lesions and occlusal factors determined by using pressure-detecting sheet. J. Dent..

[12] Guimarães JC (2014). Stress amplifications in dental non-carious cervical lesions. J. Biomech..

[13] Lubisich EB, Hilton TJ, Ferracane J (2010). Cracked teeth: a review of the literature. J. Esthet. Restor. Dent..

[14] Sailer I, Gottner J, Känel S, Franz Hämmerle CH (2009). Randomized controlled clinical trial of zirconia-ceramic and metal-ceramic posterior fixed dental prostheses: a 3-year follow-up. Int. J. Prosthodont..

[15] Dejak B, Młotkowski A (2015). A comparison of stresses in molar teeth restored with inlays and direct restorations, including polymerization shrinkage of composite resin and tooth loading during mastication. Dent. Mater..

[16] Ausiello P (2017). Mechanical behavior of bulk direct composite versus block composite and lithium disilicate indirect Class II restorations by CAD-FEM modeling. Dent. Mater..

[17] Soliman S (2016). Influence of cavity margin design and restorative material on marginal quality and seal of extended class II resin composite restorations in vitro. J. Adhes. Dent..

[18] Ausiello P (2017). The effects of cavity-margin-angles and bolus stiffness on the mechanical behavior of indirect resin composite class II restorations. Dent. Mater..

[19] Pascalea A, Rugeb S, Hauthc S, Kordaßd B, Linsene L (2015). Chewing simulation with a physically accurate deformable model Kausimulation mit einem physikalisch exakten verformbaren Modell. Int. J. Comput. Dent..

[20] Gibbs CH (1981). Occlusal forces during chewing and swallowing as measured by sound transmission. J. Prosthet. Dent..

[21] Desai PD, Das UK (2011). Comparison of fracture resistance of teeth restored with ceramic inlay and resin composite: An in vitro study. Indian J. Dent. Res..

[22] Wang M, Mehta N (2013). A possible biomechanical role of occlusal cusp–fossa contact relationships. J. Oral. Rehabil..

[23] Liu X (2017). Proprioceptive mechanisms in occlusion‐stimulated masseter hypercontraction. Eur. J. Oral. Sci..

[24] Lund JP, Kolta A (2006). Generation of the central masticatory pattern and its modification by sensory feedback. Dysphagia.

[25] Wang XR, Zhang Y, Xing N, Xu YF, Wang MQ (2013). Stable tooth contacts in intercuspal occlusion makes for utilities of the jaw elevators during maximal voluntary clenching. J. Oral. Rehabil..

[26] Sharma A (2013). History of materials used for recording static and dynamic occlusal contact marks: a literature review. J. Clin. Exp. Dent..

[27] WANG YL (2011). Patterns and forces of occlusal contacts during lateral excursions recorded by the T‐Scan II system in young Chinese adults with normal occlusions. J. Oral. Rehabil..

[28] Al-Nimri KS, Bataineh AB, Abo-Farha S (2010). Functional occlusal patterns and their relationship to static occlusion. Angle Orthod..

[29] Weissman-Fogel I (2011). Abnormal cortical activity in patients with temporomandibular disorder evoked by cognitive and emotional tasks. Pain.

[30] Tröltzsch M, Cronin R, Brodine A, Frankenberger R, Messlinger K (2011). Prevalence and association of headaches, temporomandibular joint disorders, and occlusal interferences. J. Prosthet. Dent..

[31] Fujii T (2003). The relationship between the occlusal interference side and the symptomatic side in temporomandibular disorders. J. Oral. Rehabil..

[32] Shiga H, Kobayashi Y, Arakawa I, Yokoyama M, Tanaka A (2009). Relationship between pattern of masticatory path and state of lateral occlusal contact. J. Oral. Rehabil..

[33] Zhang Y-R, Du W, Zhou X-D, Yu H-Y (2014). Review of research on the mechanical properties of the human tooth. Int. J. Oral. Sci..

[34] Oladapo BI, Zahedi SA, Vahidnia F, Ikumapayi O, Farooq MU (2018). Three-dimensional finite element analysis of a porcelain crowned tooth. Beni Suef Univ. J. Basic Appl. Sci..

[35] Benazzi S, Kullmer O, Grosse IR, Weber GW (2011). Using occlusal wear information and finite element analysis to investigate stress distributions in human molars. J. Anat..

[36] Zhang H, Cui JW, Lu X, Wang MQ (2017). Finite element analysis on tooth and periodontal stress under simulated occlusal loads. J. Oral. Rehabil..

[37] Phanachet I (2003). Functional heterogeneity in the superior head of the human lateral pterygoid. J. Dent. Res..

[38] Chen H, Whittle T, Gal J, Murray G, Klineberg I (2017). The medial pterygoid muscle: a stabiliser of horizontal jaw movement. J. Oral. Rehabil..

[39] Benazzi S, Kullmer O, Grosse IR, Weber GW (2012). Brief communication: comparing loading scenarios in lower first molar supporting bone structure using 3D finite element analysis. Am. J. Phys. Anthropol..

[40] Mesnard M (2011). Biomechanical analysis comparing natural and alloplastic temporomandibular joint replacement using a finite element model. J. Oral. Maxillofac. Surg..

[41] Guo S (2017). Interferential effect of the over-erupted third molar on chewing movement. Arch. Oral. Biol..

[42] Lafontaine N, Rossi R, Cervera M, Chiumenti M (2015). Explicit mixed strain-displacement finite element for dynamic geometrically non-linear solid mechanics. Comput. Mech..

[43] Ortega R, Orden JCG, Cruchaga M, García C (2017). Energy-consistent simulation of frictional contact in rigid multibody systems using implicit surfaces and penalty method. Multibody Syst. Dyn..

[44] Bhattacharya P, Betts D, van Lenthe GH (2018). A novel contact interaction formulation for voxel‐based micro‐finite‐element models of bone. Int. J. Numer. Methods Eng..

[45] Saini H (2020). Occlusal load modelling significantly impacts the predicted tooth stress response during biting: a simulation study. Computer Methods Biomech. Biomed. Eng..

[46] Palamara D, Palamara J, Tyas M, Messer H (2000). Strain patterns in cervical enamel of teeth subjected to occlusal loading. Dent. Mater..

[47] Dejak B, Młotkowski A, Romanowicz M (2003). Finite element analysis of stresses in molars during clenching and mastication. J. Prosthet. Dent..

[48] Rues S, Huber G, Rammelsberg P, Stober T (2011). Effect of impact velocity and specimen stiffness on contact forces in a weight-controlled chewing simulator. Dent. Mater..

[49] Mamoun JS, Napoletano D (2015). Cracked tooth diagnosis and treatment: an alternative paradigm. Eur. J. Dent..

[50] Barani A, Chai H, Lawn BR, Bush MB (2015). Mechanics analysis of molar tooth splitting. Acta Biomater..

[51] DeLong R (2006). Intra-oral restorative materials wear: rethinking the current approaches: how to measure wear. Dent. Mater..

[52] Julià-Sánchez S, Álvarez-Herms J, Cirer-Sastre R, Corbi F, Burtscher M (2020). The influence of dental occlusion on dynamic balance and muscular tone. Front. Physiol..

[53] Wu D, Liu J (2019). Occlusal interference induces oxidative stress and increases the expression of UCP3 in the masseter muscle: a rat model. Arch. Oral. Biol..

[54] Zhang H-Y (2021). Masseter response to long-term experimentally induced anterior crossbite in Sprague-Dawley rats. Arch. Oral. Biol..

[55] Zhang H-Y (2020). Injury responses of Sprague-Dawley rat jaw muscles to an experimental unilateral anterior crossbite prosthesis. Arch. Oral. Biol..

[56] Achour T, Merdji A, Bouiadjra BB, Serier B, Djebbar N (2011). Stress distribution in dental implant with elastomeric stress barrier. Mater. Des..

[57] Liu C-W, Chang Y-M, Shen Y-F, Hong H-H (2015). Using the T-scan III system to analyze occlusal function in mandibular reconstruction patients: a pilot study. Biomed. J..

[58] Becker, I. M. *Comprehensive Occlusal Concepts in Clinical Practice* (John Wiley & Sons, 2010).

[59] Mulvihill DM, Kartal ME, Nowell D, Hills DA (2011). An elastic–plastic asperity interaction model for sliding friction. Tribology Int..

