# Supplementary material for: Development of a biomechanical model for dynamic occlusal stress analysis
Source: Int J Oral Sci. 2021 Sep 8;13:29. doi: 10.1038/s41368-021-00133-5 (PMC8423745; doi:10.1038/s41368-021-00133-5)
Supplement: Supplementary file 1 — supplemental Table+1-4 [file 41368_2021_133_MOESM1_ESM.docx]

Supplemental Table 1. The EGN recordings of the centric closing task

| time (s) | Z (mm) | Y (mm) | X (mm) |
| --- | --- | --- | --- |
| 0 | -0.45 | 0.11 | 0.18 |
| 0.01 | -0.41 | 0.13 | 0.19 |
| 0.02 | -0.38 | 0.18 | 0.24 |
| 0.03 | -0.3 | 0.28 | 0.25 |
| 0.04 | -0.21 | 0.37 | 0.23 |
| 0.05 | -0.15 | 0.54 | 0.26 |
| 0.06 | -0.06 | 0.67 | 0.27 |
| 0.07 | 0.03 | 0.85 | 0.28 |
| 0.08 | 0.11 | 1.02 | 0.31 |
| 0.09 | 0.24 | 1.18 | 0.27 |
| 0.1 | 0.35 | 1.41 | 0.33 |
| 0.11 | 0.51 | 1.67 | 0.33 |
| 0.12 | 0.67 | 1.97 | 0.37 |
| 0.13 | 0.78 | 2.3 | 0.34 |
| 0.14 | 0.91 | 2.69 | 0.38 |
| 0.15 | 1.04 | 3.21 | 0.46 |
| 0.16 | 1.17 | 3.74 | 0.52 |
| 0.17 | 1.33 | 4.37 | 0.63 |
| 0.18 | 1.46 | 5.1 | 0.76 |

Supplemental Table 2. The EGN recordings of the centric to protrusion task

| time (s) | Z (mm) | Y (mm) | X (mm) |
| --- | --- | --- | --- |
| 0 | 0.45 | -0.11 | -0.18 |
| 0.01 | 0.43 | -0.15 | -0.2 |
| 0.02 | 0.45 | -0.15 | -0.21 |
| 0.03 | 0.44 | -0.18 | -0.22 |
| 0.04 | 0.42 | -0.16 | -0.22 |
| 0.05 | 0.42 | -0.19 | -0.23 |
| 0.06 | 0.42 | -0.19 | -0.26 |
| 0.07 | 0.38 | -0.22 | -0.27 |
| 0.08 | 0.42 | -0.17 | -0.24 |
| 0.09 | 0.41 | -0.17 | -0.23 |
| 0.1 | 0.42 | -0.17 | -0.25 |
| 0.11 | 0.43 | -0.17 | -0.23 |
| 0.12 | 0.41 | -0.16 | -0.24 |
| 0.13 | 0.42 | -0.17 | -0.23 |
| 0.14 | 0.43 | -0.18 | -0.26 |
| 0.15 | 0.42 | -0.15 | -0.2 |
| 0.16 | 0.43 | -0.15 | -0.22 |
| 0.17 | 0.42 | -0.13 | -0.18 |
| 0.18 | 0.41 | -0.13 | -0.21 |
| 0.19 | 0.4 | -0.14 | -0.2 |
| 0.2 | 0.38 | -0.12 | -0.21 |
| 0.21 | 0.38 | -0.18 | -0.24 |
| 0.22 | 0.4 | -0.17 | -0.21 |
| 0.23 | 0.41 | -0.18 | -0.13 |
| 0.24 | 0.45 | -0.27 | -0.1 |
| 0.25 | 0.45 | -0.36 | 0.01 |
| 0.26 | 0.44 | -0.48 | 0.1 |
| 0.27 | 0.39 | -0.65 | 0.19 |
| 0.28 | 0.28 | -0.79 | 0.3 |
| 0.29 | 0.19 | -0.98 | 0.38 |
| 0.3 | 0.09 | -1.06 | 0.53 |
| 0.31 | -0.03 | -1.13 | 0.75 |
| 0.32 | -0.12 | -1.25 | 0.88 |
| 0.33 | -0.22 | -1.34 | 1.1 |
| 0.34 | -0.27 | -1.4 | 1.26 |
| 0.35 | -0.33 | -1.46 | 1.45 |
| 0.36 | -0.37 | -1.49 | 1.61 |
| 0.37 | -0.38 | -1.54 | 1.76 |
| 0.38 | -0.37 | -1.57 | 1.86 |
| 0.39 | -0.37 | -1.62 | 1.95 |
| 0.4 | -0.33 | -1.66 | 2.01 |
| 0.41 | -0.31 | -1.71 | 2.06 |
| 0.42 | -0.28 | -1.73 | 2.1 |
| 0.43 | -0.22 | -1.76 | 2.14 |
| 0.44 | -0.16 | -1.69 | 2.21 |
| 0.45 | -0.12 | -1.68 | 2.23 |
| 0.46 | -0.09 | -1.64 | 2.24 |
| 0.47 | -0.1 | -1.61 | 2.26 |
| 0.48 | -0.07 | -1.57 | 2.26 |
| 0.49 | -0.07 | -1.52 | 2.29 |
| 0.5 | -0.03 | -1.43 | 2.32 |
| 0.51 | -0.01 | -1.41 | 2.31 |
| 0.52 | -0.02 | -1.36 | 2.3 |
| 0.53 | 0 | -1.34 | 2.31 |
| 0.54 | 0.03 | -1.29 | 2.33 |
| 0.55 | 0.04 | -1.25 | 2.38 |
| 0.56 | 0.09 | -1.19 | 2.43 |
| 0.57 | 0.1 | -1.15 | 2.49 |
| 0.58 | 0.13 | -1.12 | 2.54 |
| 0.59 | 0.16 | -1.14 | 2.56 |
| 0.6 | 0.19 | -1.11 | 2.62 |
| 0.61 | 0.23 | -1.13 | 2.66 |

Supplemental Table 3. The EGN recordings of the centric to left lateral extension task

| time(s) | Z(mm) | Y(mm) | X(mm) |
| --- | --- | --- | --- |
| 0 | 0.1 | 0.41 | 0.4 |
| 0.01 | 0.13 | 0.43 | 0.4 |
| 0.02 | 0.15 | 0.45 | 0.39 |
| 0.03 | 0.21 | 0.51 | 0.37 |
| 0.04 | 0.36 | 0.65 | 0.34 |
| 0.05 | 0.58 | 0.87 | 0.32 |
| 0.06 | 0.84 | 1.14 | 0.29 |
| 0.07 | 1.19 | 1.47 | 0.26 |
| 0.08 | 1.66 | 1.83 | 0.25 |
| 0.09 | 2.16 | 2.04 | 0.17 |
| 0.1 | 2.61 | 2.09 | -0.03 |
| 0.11 | 3.01 | 2.04 | -0.22 |
| 0.12 | 3.31 | 1.97 | -0.39 |
| 0.13 | 3.48 | 1.89 | -0.54 |
| 0.14 | 3.59 | 1.84 | -0.65 |
| 0.15 | 3.66 | 1.82 | -0.72 |
| 0.16 | 3.67 | 1.81 | -0.79 |
| 0.17 | 3.67 | 1.82 | -0.81 |
| 0.18 | 3.7 | 1.85 | -0.79 |
| 0.19 | 3.69 | 1.86 | -0.78 |
| 0.2 | 3.68 | 1.86 | -0.8 |
| 0.21 | 3.72 | 1.87 | -0.81 |
| 0.22 | 3.79 | 1.9 | -0.81 |
| 0.23 | 3.87 | 1.9 | -0.83 |
| 0.24 | 3.93 | 1.89 | -0.87 |
| 0.25 | 4 | 1.89 | -0.9 |
| 0.26 | 4.06 | 1.89 | -0.92 |
| 0.27 | 4.09 | 1.9 | -0.91 |
| 0.28 | 4.1 | 1.9 | -0.91 |

Supplemental Table 4. The EGN recordings of the centric to right lateral extension task

| time(s) | Z(mm) | Y(mm) | X(mm) |
| --- | --- | --- | --- |
| 0 | -0.07 | 0.18 | 0.1 |
| 0.01 | -0.31 | 0.24 | 0.13 |
| 0.02 | -0.61 | 0.3 | 0.15 |
| 0.03 | -0.96 | 0.43 | 0.18 |
| 0.04 | -1.32 | 0.56 | 0.19 |
| 0.05 | -1.71 | 0.68 | 0.17 |
| 0.06 | -2.07 | 0.85 | 0.19 |
| 0.07 | -2.42 | 1.04 | 0.18 |
| 0.08 | -2.77 | 1.26 | 0.2 |
| 0.09 | -3.11 | 1.56 | 0.25 |
| 0.1 | -3.45 | 1.88 | 0.33 |
| 0.11 | -3.76 | 2.16 | 0.34 |
| 0.12 | -4.02 | 2.48 | 0.44 |
| 0.13 | -4.28 | 2.8 | 0.53 |
| 0.14 | -4.49 | 3.09 | 0.64 |
| 0.15 | -4.69 | 3.36 | 0.73 |
| 0.16 | -4.91 | 3.58 | 0.82 |
| 0.17 | -5.11 | 3.88 | 0.95 |
| 0.18 | -5.34 | 4.15 | 1.07 |
| 0.19 | -5.57 | 4.45 | 1.17 |
| 0.2 | -5.74 | 4.76 | 1.31 |
| 0.21 | -5.97 | 5.05 | 1.42 |
| 0.22 | -6.15 | 5.38 | 1.62 |
| 0.23 | -6.29 | 5.67 | 1.75 |
| 0.24 | -6.36 | 5.87 | 1.87 |
| 0.25 | -6.37 | 6.08 | 1.98 |
| 0.26 | -6.37 | 6.2 | 2.06 |
| 0.27 | -6.37 | 6.35 | 2.16 |
| 0.28 | -6.38 | 6.43 | 2.19 |
| 0.29 | -6.38 | 6.52 | 2.25 |
| 0.3 | -6.38 | 6.59 | 2.3 |
| 0.31 | -6.38 | 6.62 | 2.31 |
| 0.32 | -6.39 | 6.66 | 2.36 |
| 0.33 | -6.96 | 6.67 | 2.38 |
| 0.34 | -6.96 | 6.69 | 2.39 |
